# Surgical site infection after intracorporeal anastomosis for left-sided colon cancer: study protocol for a non-inferiority multicenter randomized controlled trial (STARS)

**DOI:** 10.1186/s13063-022-06914-5

**Published:** 2022-11-22

**Authors:** Liang He, Meng Li, Jia-xin Zhang, Wei-hua Tong, Yan Chen, Quan Wang

**Affiliations:** Department of Gastric and Colorectal Surgery, General Surgery Center, The First Hospital of Jilin University, Changchun, China

**Keywords:** Surgical site infection, Laparoscopic surgery, Intracorporeal anastomosis, Randomized controlled trial

## Abstract

**Background:**

Surgical site infection (SSI), as one of the most common hospital-acquired infection, is usually associated with increased morbidity, mortality, and health care burden. SSI is a significant perioperative complication after colon cancer surgery, particularly for left-sided colon cancer. This paper describes the background and design of the “Surgical Site Infection after intracorporeal anastomosis for Left-sided Colon Cancer: study protocol for a non-inferiority multicenter Randomized Controlled Trial (STARS).” The STARS trial aims to compare the incidence of SSI after intracorporeal anastomosis and extracorporeal anastomosis after radical resection of colon cancer and to explore the risk factors of SSI.

**Methods:**

A total of 354 left colon cancer patients from 8 hospitals in China will be enrolled in this multi-center randomized controlled study. The primary outcome of this study is the incidence of SSI 30 days after left-sided colon cancer surgery. Secondary outcome measures include operation time, blood loss, conversion rate, incidence of perioperative complications, completeness of resection, number of lymph nodes collected and postoperative recovery characteristics, 3-year disease-free survival, and 5-year overall survival. The first patient was enrolled in January 2021.

**Discussion:**

To our knowledge, this is the first prospective multicenter study to investigate whether there is a difference in the SSI incidence after intracorporeal and extracorporeal anastomosis for left-sided colon cancer in China. The results may provide more evidence that supports performing total laparoscopic left-sided colon cancer surgery.

**Trial registration:**

The trial has been registered on ClinicalTrials.gov website (ID: NCT04201717). Registered on September 22, 2020

**Supplementary Information:**

The online version contains supplementary material available at 10.1186/s13063-022-06914-5.

## Administrative information

**Table:** 

Title{1}	Surgical Site Infection after intracorporeal anastomosis for Left-sided Colon Cancer: study protocol for a non-inferiority multicenter Randomized Controlled Trial (STARS)
Trial registration {2a and 2b}	ClinicalTrials.gov Identifier: NCT04201717; Registered September 22, 2020
Protocol version {3}	Study Protocol v1.0, 20 December 2019
Funding {4}	This study was funded by the Development Center for Medical Science & Technology National Health Commission of the People’s Republic of China (W2017ZWS01 and WA2021RW19).
Author details {5a}	^1^Department of Gastric and Colorectal Surgery, General Surgery Center, The First Hospital of Jilin University
Name and contact information for the trial sponsor {5b}	Investigator initiated trial; Quan Wang (Principal Investigator) wquan@jlu.edu.cn
Role of sponsor {5c}	This is an investigator initiated trial. The funders had no role in the design of the study and data collection, analysis, interpretation of data, manuscript writing, nor the decision to submit the report for publication.

## Background

It is estimated that there were more than 1.09 million new cases of colon cancer and more than 500,000 deaths from colon cancer in 2018. In developed countries, the incidence of colon cancer is higher than in developing countries. Usually, cancers proximal to the splenic flexure are defined as right-sided and cancers at or distal to the splenic flexure are defined as left-sided [1]. It is reported that left-sided colon cancer has different clinical and biological characteristics from right-sided colon cancer [2] and has a more favorable prognosis than the latter [3].

Although neoadjuvant therapy and postoperative chemotherapy had been demonstrated to reduce the risk of tumor recurrence and death [4, 5], surgery remains the main treatment for patients with potentially curable colon cancer [6]. Currently, main surgical methods include traditional open surgery, minimally invasive surgery using laparoscopic techniques, and emerging robotic surgery. Compared with open surgery, laparoscopic surgery has achieved similar oncological outcomes in colon cancer [7] or left-sided colon cancer [8]. As for overall survival, laparoscopic surgery has been demonstrated oncologically safe, and was non-inferior to open surgery for patients with stage II or III colon cancer [9, 10].

However, surgical site infection (SSI) is one of the most common nosocomial infection in colonic surgery, with an incidence that varies between 5 and 30% [11, 12]. SSI is usually split into superficial incisional SSI, deep incisional SSI, and organ/space SSI [13, 14]. Data from the American College of Surgeons National Surgical Quality Improvement Program showed the overall SSI rate was 12.3%, and left resections had increased odds of superficial SSI compared with right resections [15]. There is an urgent need to determine the incidence of SSI, as well as strategies to reduce SSI rate [16].

The aim of STARS trial is to evaluate the incidence of SSI and safety of the intracorporeal anastomosis after left-sided colon surgery. In this multi-center study, the participating surgeons have already overcome their learning curve and their laparoscopies had been video documented and analyzed in a blinded fashion beforehand by independent experts.

## Methods/design

### Study design

**Fig. 1:**
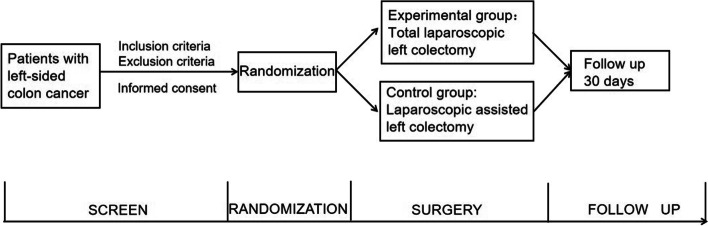
The study design flowchart

This is a prospective multicenter randomized controlled trial to evaluate the incidence of SSI and safety of intracorporeal anastomosis compared to extracorporeal anastomosis for left-sided colon cancer (see Fig. 1). A total of 354 left-sided colon cancer patients will be enrolled and randomly assigned to two groups through central randomization in a 1:1 ratio. Eight participating centers from China will complete the recruitment of participants. We followed the standardized program intervention: Standard Protocol Item Recommendations for Interventional Trials (SPIRIT) 2013 [17].

## Participants

### Inclusion criteria

The patients will be included if they meet the following 7 items: (1) age between 18 and 80 years old (male or female); (2) histologically or cytologically diagnosed as left-sided colon cancer (distal transverse colon, left colic flexure, descending colon or sigmoid colon); (3) clinical stage T1–4a, N0–2, and M0; (4) Eastern Cooperative Oncology Group (ECOG) performance status ≤ 2; (5) suitable for elective laparoscopic surgical resection; (6) no previous systemic chemotherapy or radiotherapy; and (7) willing to provide written informed consent and willing to follow the research program.

### Exclusion criteria

The patients will be excluded if they match any of the following 9 items: (1) cardiopulmonary dysfunction (NYHA cardiac function classification II-IV), liver dysfunction (MELD score greater than 12), or kidney dysfunction (serum creatinine above the upper limit of normal); (2) metastatic or multiple carcinoma; (3) patients with bowel obstruction, bowel perforation, or bowel bleeding requiring emergency surgery; (4) be accompanied with severe psychiatric illness; (5) be pregnant or during lactation; (6) fail to control diabetes mellitus (6.1–8.3 mmol/L); (7) patients with a history of taking hormone drugs; (8) planned synchronous abdominal organ resections; and (9) end-to-end anastomosis.

### Discontinuation criteria

Participants can decide to withdraw from the study at any time for their own reasons. Investigators, on the other hand, can exclude patients who meet the following criteria: (1) after enrollment, patients who need emergency surgical resection due to intestinal obstruction, intestinal perforation, or intestinal bleeding; (2) patients with distant metastases confirmed by intraoperative exploration or postoperative pathology; (3) combined organ resection is required after intraoperative exploration; and (4) serious adverse events occurred preoperatively (according to the Common Terminology Criteria for Adverse Events, CTCAE version 5.0). The corresponding data of patients who withdrew from the study will not be reused, and the patients will not be allowed to re-enter the study.

### Recruitment and trial timeline

Participants will be screened and recruited from the outpatient and inpatient departments of study sites from November 2019 to December 2021. Patients screened out will be assigned a unique identification number. The first patient was enrolled in January 2021.

## Randomization

Randomization will be conducted immediately after recruitment. To guarantee randomization concealment adequately, and not be influenced either by the surgeon or the participants, randomization will be performed by a researcher who is not participating in this study from the Research Centre of Clinical Epidemiology, Jilin University First Hospital. The random codes will be designed in a 1:1 ratio (experimental group or control group) using the R (version 3.3.3, University of Auckland, Oakland, New Zealand). The random codes will be sealed in an opaque envelope and handed to the researcher responsible for grouping. After unified training, the site investigators can apply for randomization through text messages, e-mail, or telephone application. When grouping, the envelope with the unique identification number for the participant will be opened, and the group assignment will immediately be delivered to the researcher of the study site.

## Blinding

The clinical surgeons, outcome assessors, data recorders, and statisticians will all operate independently. Due to the nature of the surgical interventions, the surgeons will not be blinded to treatment allocation. However, the investigators who assess or analyze the end point and the patients will be blinded to group allocation to reduce the risk of bias. Unblinding will not be performed unless the finalization of the main data analysis or required for patient’s safety. After the data analysis, we will have a blinded interpretation of the study results to minimize misleading data interpretation.

## Interventions/procedures

### Preparation procedures

All patients will routinely drink the solution of polyethylene glycol electrolytes powder for bowel preparation the evening before operation. Oral antibiotic (metronidazole 800 mg and gentamycin sulfate 160 mg) will be given the day before surgery. Mezlocillin sodium will be administered as prophylactic antibiotic 20 min before the anesthetic induction. A urinary catheter will be inserted after anesthetic induction.

### Surgical techniques

#### General surgical procedures

**Fig. 2:**
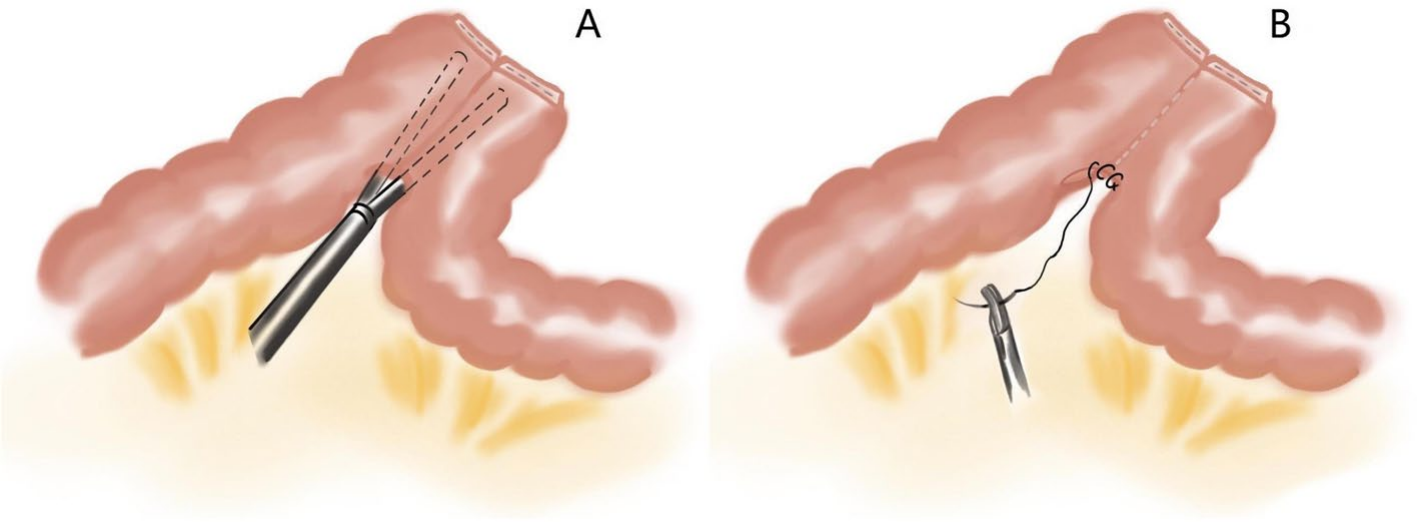
**A** Two ends of a 60-mm linear laparoscopic stapler are inserted into the colons through enterotomies separately, then close along the antimesenteric borders and fire. **B** The common enterotomy is sutured continuously

The patients will be placed in supine position with legs spread. The surgeon and assistant will stand on the right side of the operating bed. The initial port (10mm) will be placed above the umbilicus by using the open technique. Four other ports will be placed separately under laparoscopic guidance (positions are shown in the Fig. 2). Complete mesocolic excision (CME) and central vascular ligation principles will be applied in all cases. We will use a medial to lateral approach. Mesenteric vessels (inferior mesenteric vein, left colic artery, 1-2 sigmoid arteries) will be exposed and divided at their origins with absorbable or non-absorbable clips. The greater omentum will be separated from the transverse colon by using the harmonic ace. The splenic flexure and descending colon will be mobilized [18, 19].

#### The control group procedures

The surgeon will use wound edge protectors to take out the colon through a small incision in the middle of the abdomen or the outer edge of the left rectus abdominis. We will use a ruler and methylthionine chloride solution to mark the range of colon resection (10 cm from tumor). Guiding by the marker, the marginal vessels and mesentery will be divided extracorporeally. Methods of anastomosis will be selected by preferences of surgeons. The drainage will be placed.

#### The experiment group procedure (also see Supplementary 1)

**Fig. 3:**
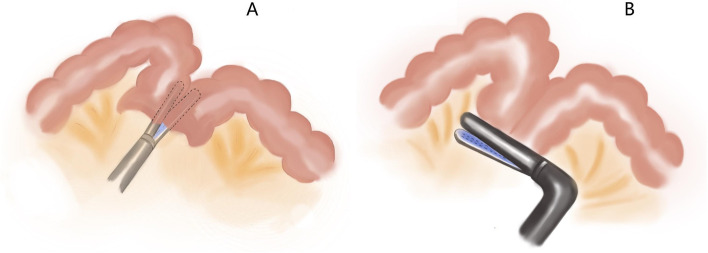
**A** Two ends of 60-mm linear laparoscopic stapler are inserted into the colons through enterotomies separately, then close along the antimesenteric borders and fire. **B** The common enterotomy is closed by a reload 60-mm linear laparoscopic stapler perpendicular to colon ends

**Fig. 4:**
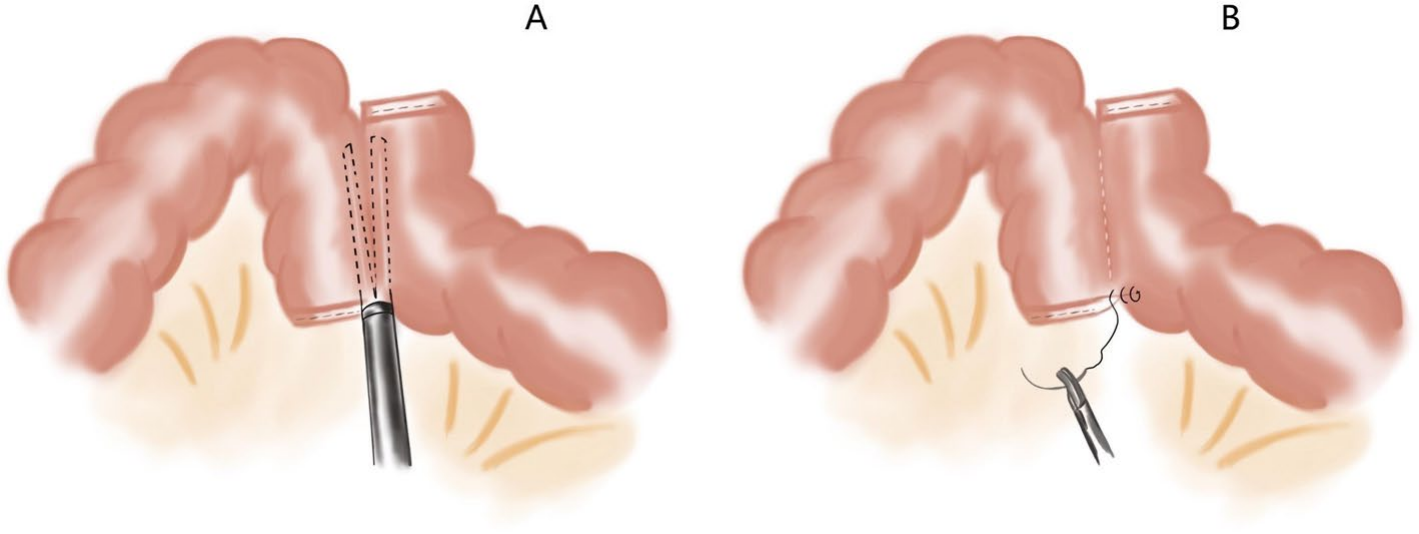
**A** Two ends of 60-mm linear laparoscopic stapler are inserted into the colons through the enterotomies separately, then close along the antimesenteric borders and fire. **B** The common enterotomy is sutured continuously

We will use a flexible scale and methylthionine chloride solution to mark the range of colon resection (10 cm from tumor). Guiding by the marker, the marginal vessels and mesentery will be divided by intracorporeally. The proximal and distal colons will be resected intracorporeally by using the 60mm linear laparoscopic stapler. The specimen was placed anterior to the spleen until the end of anastomosis. Anti/iso-peristaltic side-to-side or overlap method anastomosis could be applied [20, 21]. We list three intracorporeal methods of anastomosis (Figs. 2, 3, and 4). The specimen will be placed into a sterile plastic bag and retrieved through a small midline or suprapubic incision. The drainage will be placed.

##### Conventional isoperistaltic side-to-side anastomosis

On antimesenteric borders, two small enterotomies are made about 8 cm from the colonic ends. Both colonic ends are placed on the upper left abdomen. Two ends of a 60mm linear laparoscopic stapler are inserted into the colons through enterotomies separately, then close along the antimesenteric borders and fire (Fig. 2A). The 3-0 knotless absorbable suture is used to suture the common enterotomy continuously (Fig. 2B).

##### Antiperistaltic side-to-side anastomosis

On antimesenteric borders, two small enterotomies are made on the colon ends. Both colon segment ends are placed near the abdomen midline. Two ends of 60-mm linear laparoscopic stapler are inserted into the colons through enterotomies separately, then close along the antimesenteric borders and fire (Fig. 3A). The common enterotomy is closed by a reload 60-mm linear laparoscopic stapler perpendicular to colon ends (Fig. 3B) or by suturing the common enterotomy continuously with the 3-0 knotless absorbable suture.

##### Overlap method anastomosis

According to the tumor location and convenience of the anastomosing, the linear laparoscopic stapler can be placed into the abdominal cavity through cephalic or caudal trocars. If the transverse colon end is with low mobility, the caudal approach to insert the stapler will be much more convenience (Fig. 4A). In this approach, one small enterotomy is made at the antimesenteric borders of the transverse colonic end, while another enterotomy is made about 8cm from the descending or sigmoid colonic end. A 60 mm linear laparoscopic stapler is placed in by caudal trocars, and two ends are inserted into the colons through the enterotomies separately, then closed along the antimesenteric borders and fire (Fig. 4B). The 3-0 knotless absorbable suture used to suture the common enterotomy continuously. In the cephalic approach, the position of two enterotomies on colons are be made just opposite to the caudal cases.

## Outcomes

### Primary outcome

In this study, the primary outcome is the incidence of SSI within 30 days after surgery, which is diagnosed according to the CDC guidelines (also see Supplementary 2) [22]. Based on CDC’s diagnostic criteria, SSI is divided into three types: superficial incisional SSI, deep incisional SSI, and organ/space SSI.

### Secondary outcomes

The secondary outcomes include the following: (1) skin to skin time (min); (2) total blood loss (ml); (3) conversion rate (to laparoscopic assisted surgery or open surgery); (4) incidence of perioperative complications; (5) completeness of resection (R0 resection); (6) number of lymph nodes dissected; (7) postoperative recovery: time to first flatus, time to first defecation, time to food intake, and hospitalization days; (8) length of incision (cm); (9) visual analog score (VAS) of pain 1–3 days after operation; and (10) 3-year disease-free survival and 5-year overall survival.

### Data collection and follow-up

The outcome assessors are trained on the CDC definitions of SSI to produce good inter-rater reliability for the assessment of SSI. Medical records of participants enrolled will be completed and saved to preserve related data. The time point of the study and assessments are given in Table 1. Each participant will be followed until recurrence or death over a period of 5 years after the scheduled surgery. All patients are required to take an abdominal and pelvic CT scan 30 days after the surgery, which is part of the routine practice. If the CT result shows evidence of SSI, the radiologist and surgeon will comprehensively evaluate whether SSI has occurred. Appropriate strategies will be used to improve patient adherence and ensure data integrity. All participants will be informed of the study procedures and potential benefits and risks when they sign the informed consent form to make them fully understand the inconvenience and significance of data collection. In addition, patients after abdominal surgery need to come back to our hospital for other treatment of combined injuries including suture removal. Our medical team will provide standard post-operative care for the enrolled participants to promote their adherence. Investigators will contact the patients 1 week before the follow-up session to enhance the attendance rate. Patients who default a scheduled appointment will be contacted to re-arrange another appointment within 1 week. Telephone follow-up calls, WeChat group discussion, and personal interviews will be used for maximizing retention and complete follow-up.

**Table 1:** The time point and assessments of the STARS study

Time point	Study period							
	Screening	Treatment	Follow-up (month)					
			1	12	24	36	48	60
Informed consent	√							
Inclusion criteria	√							
Exclusion criteria	√	√	√	√	√	√	√	√
Baseline data	√							
Blood test	√							
Tumor marker test	√							
Chest CT	√							
Pelvic MRI	√							
Abdominal CT	√		√					
Electrocardiogram	√							
Colonoscopy	√							
Surgery		√						
Complications		√	√					
Recurrence/death			√	√	√	√	√	√

### Sample size calculation

This study is multicenter, randomized, controlled, non-inferiority trial for patients aged 18–80 years old with left-sided colon cancer. According to the previous data of our single center, the incidence of SSI after extracorporeal anastomosis left colon cancer surgery was 23.3%, and the incidence of SSI after intracorporeal anastomosis was 16.7%. Intracorporeal anastomosis is believed to be inferior to extracorporeal anastomosis when there is a difference in SSI rate of more than 5% 30 days after surgery [23]. Therefore, sample size is calculated based on non-inferiority with a difference of 5% with a one-sided level of significance of 0.025, a ratio of 1:1, a power of 0.80 and a 20% drop-out rate. A total of 354 patients is needed, 177 patients in the experiment group and 177 patients in the control group.

### Statistical analysis

Statistical analysis will be carried out using R (version 4.0.0, University of Auckland, Oakland, New Zealand). The baseline characteristics will be presented as mean ± standard deviations (for normally distributed continuous variables), median and interquartile range (for skewed continuous variables) or frequency (for categorical variables). The *t* test, Wilcoxon rank sum test, or Rao-scott-*χ*^2^ test, as appropriate, will be used to explore difference by groups. A two-tailed *P*-value < 0.05 will be considered significant (unless otherwise specified). Intention-to-treat analysis includes participants who have completed randomization. In per-protocol population analysis, enrolled participants who have completed the study contents will be analyzed. And the results between intention-to-treat analysis and per-protocol population analysis will be compared. We have no imputation plans for missing data, and no plans for interim analysis.

### Participating centers

Eight centers will participate in this study: First Hospital of Jilin University; West China Hospital, Sichuan University; Fudan University Shanghai Cancer Center; Nanfang Hospital of Southern Medical University; Peking University Cancer Hospital; China-Japan Union Hospital of Jilin University; Ruijin Hospital, Shanghai Jiaotong University School of Medicine; and Beijing Friendship Hospital, Capital Medical University.

### Safety and reporting of serious adverse events

In this study, all adverse events from the beginning to the end of the study will be recorded in the case report form carefully. Serious adverse events such as death or life-threatening events will be reported to the principal investigator immediately. All adverse events will be carefully monitored, managed, and tracked in a timely and completely manner until they are properly resolved, stabilized, or returned to normal.

### Data management

All relevant clinical data including demographic and medical information will be collected from the electronic medical record. The collected data will be encoded to protect the privacy of all participants and then be entered into an electronic case report forms (eCRFs) by trained research assistants. Double data entry will be performed to ensure that the data collected are accurate and verifiable from source documents. Input rules including field and range checks will also be set to minimize data entry errors. The research coordinator will periodically check the data distribution, verify outliers, and track missing data to improve data integrity and data validation. Data storage and backup will be managed by the edocdata platform (edocdata.com), which will track all changes to the data and retains a history for each variable. Unauthorized persons or institutions will not be able to access any data stored on this server. Both paper and electronic documents will be preserved for at least 5 years after publication.

### Data monitoring

A data monitoring committee composed of specialists in surgery and statistics was independently established to review and monitor the trial. The data monitoring committee will supervise the outcome assessments and data management, visit the participating center and verify the source data to ensure adherence to the protocol. An annual meeting will be conducted where the DMC can monitor the implementation of the entire trial, and make recommendations for continuation, discontinuation, or modification of the trial. When the trial is completed, the data will be locked and stored on edocdata platform, after which the researchers can no longer modify the data.

### Ethics and dissemination

This trial has been approved by the Ethics Review Committee of the First Hospital of Jilin University (Approval number: 19K135-001). Protocol modifications will be reviewed by the Ethics Review Committee and communicated to all participating centers. Also, the trial has been registered on ClinicalTrials.gov website (ID: NCT04201717). This trial follows the principles of the Declaration of Helsinki. All eligible patients will be informed of the study’s purpose, procedures, potential benefits, and risks. Informed consent will be obtained by the principal investigator or their sub-investigators (also see Supplementary 3-4).

At the end of the study, the findings will be presented internally at scientific conferences. The feasibility results are intended for publication in high-impact peer-reviewed journals. People who have made significant contributions to this trial will be listed as co-authors.

### Confidentiality

Participants are assigned a unique code to ensure that the participants’ anonymity is maintained. Documents containing personal information are stored separately from assessment data in locked cabinets. Except for the principal investigator (PI) and authorized research assistants, nobody will have access to the dataset unless there is an institutional or regulatory requirement. The fully anonymized experimental data are stored on secure servers of the Jilin University First Hospital and will be destroyed 5 years after the study’s conclusion.

## Discussion

Globally, SSI not only increases morbidity and mortality, but also negatively affects the life quality of patients [24]. In the intracorporeal anastomosis surgery of left colon cancer, the incidence of SSI is rarely reported. So, there is an urgent need to show the safety and feasibility of left colon cancer in SSI.

This high-quality randomized controlled study aims to investigate the incidence of SSI among patients after radical resection of left colon cancer. The results might provide us with advance clinical knowledge on the intracorporeal anastomosis left-sided colectomy and provide reference to develop better practice for prevention, diagnosis, and treatment of SSI.

## Supplementary Information

**Additional file 1.** The experiment group procedure.

**Additional file 2.** CDC guidelines.

**Additional file 3.** Informed consent in Chinese.

**Additional file 4.** Informed consent in English.

## Data Availability

Not applicable.

## Abbreviation

SSI: Surgical site infection
