# Supplementary material for: Surgical site infection after intracorporeal anastomosis for left-sided colon cancer: study protocol for a non-inferiority multicenter randomized controlled trial (STARS)
Source: Trials. 2022 Nov 22;23:954. doi: 10.1186/s13063-022-06914-5 (PMC9682838; doi:10.1186/s13063-022-06914-5)
Supplement: Supplementary file 2 — Additional file 2. CDC guidelines. [file 13063_2022_6914_MOESM2_ESM.pdf]

Table 1. Surgical Site Infection Criteria

Criterion	Surgical Site Infection (SSI)
	**Superficial incisional SSI**<br>Must meet the following criteria:
	Date of event occurs within 30 days after any NHSN operative procedure (where day 1 = the procedure date)<br>**AND**<br>involves only skin and subcutaneous tissue of the incision<br>**AND**<br>patient has at least ***one*** of the following:<br>a. purulent drainage from the superficial incision.<br>b. organism(s) identified from an aseptically-obtained specimen from the superficial incision or subcutaneous tissue by a culture or non-culture based microbiologic testing method which is performed for purposes of clinical diagnosis or treatment (for example, not Active Surveillance Culture/Testing (ASC/AST)).<br>c. superficial incision that is deliberately opened by a surgeon, physician* or physician designee and culture or non-culture based testing of the superficial incision or subcutaneous tissue is not performed<br>**AND**<br>patient has at least one of the following signs or symptoms: localized pain or tenderness; localized swelling; erythema; or heat.<br>d. diagnosis of a superficial incisional SSI by a physician* or physician designee.<br><br>* The term physician for the purpose of application of the NHSN SSI criteria may be interpreted to mean a surgeon, infectious disease physician, emergency physician, other physician on the case, or physician's designee (nurse practitioner or physician's assistant).

	Superficial Incisional SSI
**Comments**	There are two specific types of superficial incisional SSIs:<br>1. Superficial Incisional Primary (SIP) – a superficial incisional SSI that is identified in the primary incision in a patient that has had an operation with one or more incisions (for example, C-section incision or chest incision for CBGB)<br>2. Superficial Incisional Secondary (SIS) – a superficial incisional SSI that is identified in the secondary incision in a patient that has had an operation with more than one incision (for example, donor site incision for CBGB)
**Reporting Instructions for Superficial SSI**	**<u>The following do not qualify as criteria for meeting the NHSN definition of superficial incisional SSI:</u>**<br>• Diagnosis/treatment of cellulitis (redness/warmth/swelling), by itself, does not meet criterion "d" for superficial incisional SSI. Conversely, an incision that is draining or that has organisms identified by culture or non-culture based testing is not considered a cellulitis.<br>• A stitch abscess alone (minimal inflammation and discharge confined to the points of suture penetration).<br>• For an NHSN operative procedure, a laparoscopic trocar site is considered a surgical incision and not a stab wound.<br>• A localized stab wound or pin site infection is not considered an SSI; depending on the depth, these infections might be considered either a skin (SKIN) or soft tissue (ST) infection.

	**Deep incisional SSI**<br>Must meet the following criteria:
	The date of event occurs within 30 or 90 days after the NHSN operative procedure (where day 1 = the procedure date) according to the list in Table 2<br>**AND**<br>involves deep soft tissues of the incision (for example, fascial and muscle layers)<br>**AND**<br>patient has at least ***one*** of the following:<br>a. purulent drainage from the deep incision.<br>b. a deep incision that spontaneously dehisces, or is deliberately opened or aspirated by a surgeon, physician* or physician designee<br>**AND**<br>organism(s) identified from the deep soft tissues of the incision by a culture or non-culture based microbiologic testing method which is performed for purposes of clinical diagnosis or treatment (for example, not Active Surveillance Culture/Testing (ASC/AST)) or culture or non-culture based microbiologic testing method is not performed. A culture or non-culture based test from the deep soft tissues of the incision that has a negative finding does not meet this criterion.<br>**AND**<br>patient has at least ***one*** of the following signs or symptoms: fever (>38°C); localized pain or tenderness.<br>c. an abscess or other evidence of infection involving the deep incision that is detected on gross anatomical or histopathologic exam, or imaging test.<br><br>* The term physician for the purpose of application of the NHSN SSI criteria may be interpreted to mean a surgeon, infectious disease physician, emergency physician, other physician on the case, or physician's designee (nurse practitioner or physician's assistant).

Comments	Deep incisional SSI
	There are two specific types of deep incisional SSIs:<br>1. Deep Incisional Primary (DIP) – a deep incisional SSI that is identified in a primary incision in a patient that has had an operation with one or more incisions (for example, C-section incision or chest incision for CBGB)<br>2. Deep Incisional Secondary (DIS) – a deep incisional SSI that is identified in the secondary incision in a patient that has had an operation with more than one incision (for example, donor site incision for CBGB)

	**Organ/Space SSI**<br>Must meet the following criteria:
	Date of event occurs within 30 or 90 days after the NHSN operative procedure (where day 1 = the procedure date) according to the list in Table 2<br>**AND**<br>involves any part of the body deeper than the fascial/muscle layers that is opened or manipulated during the operative procedure<br>**AND**<br>patient has at least ***one*** of the following:<br>a. purulent drainage from a drain that is placed into the organ/space (for example, closed suction drainage system, open drain, T-tube drain, CT-guided drainage).<br>b. organism(s) identified from fluid or tissue in the organ/space by a culture or non-culture based microbiologic testing method which is performed for purposes of clinical diagnosis or treatment (for example, not Active Surveillance Culture/Testing (ASC/AST)).<br>c. an abscess or other evidence of infection involving the organ/space that is detected on gross anatomical or histopathologic exam, or imaging test evidence suggestive of infection.<br>**AND**<br>meets at least ***one*** criterion for a specific organ/space infection site listed in Table 3. These criteria are found in the Surveillance Definitions for Specific Types of Infections (Chapter 17)
