# Supplementary material for: Surgical site infection after intracorporeal anastomosis for left-sided colon cancer: study protocol for a non-inferiority multicenter randomized controlled trial (STARS)
Source: Trials. 2022 Nov 22;23:954. doi: 10.1186/s13063-022-06914-5 (PMC9682838; doi:10.1186/s13063-022-06914-5)
Supplement: Supplementary file 3 — Additional file 3. Informed consent in Chinese. [file 13063_2022_6914_MOESM3_ESM.pdf]

知情同意书

方案名称：全腹腔镜对比腹腔镜辅助在左半结肠癌根治术中手术部位感染的前瞻性、多中心、随机对照、非劣效临床研究

方案版本号：1.0 ，2019/12/20

知情同意书版本号：1.0 ，2019/12/20

研究机构：吉林大学第一医院

主要研究者：王权教授

患者姓名缩写：____________

您将被邀请参加一项临床研究，本知情同意书提供给您一些信息以帮助您决定是否参加此项临床研究。请您仔细阅读，如有任何疑问请向负责该项研究的有关医师提出。

您参加本项研究是自愿的。本次研究已通过吉林大学第一医院伦理委员会审查。

一、研究背景

结直肠癌是我国最常见的恶性肿瘤之一。根据最新的癌症统计信息，2015年我国结直肠癌估算新发和死亡病例分别为 37.6 万和 19.1 万，发病率和死亡率均居第 5 位。随着医学科学和技术的进步，结直肠癌的治疗已经比较成熟，形成了以手术为主的综合治疗及个体化治疗模式，TME、CME 手术原则的推广极大的规范了结直肠手术操作。III 期 COLOR II、COREAN 及 CLASICC 研究均显示腹腔镜和开放手术相比，局部复发，无疾病生存和总体生存两组间无差异。提示腹腔镜结直肠癌手术的安全性和有效性。在保证肿瘤根治效果的同时，努力减少患者的创伤一直是结直肠外科医师追求的目标。传统腹腔镜辅助手术在腔内解剖游离后仍需采取腹壁小切口辅助修剪系膜，取出标本，于腹壁外完成吻合，仍会给病人造成一定创伤，可引起术后疼痛并可导致切口感染及切口疝等并发症，而部分肥胖病人，系膜粗短，上述操作更加困难，甚至会造成系膜撕裂、出血等风险，进而削弱腹腔镜手术的微创优势。全腹腔镜左半结肠癌根治术即于腹腔镜下完成术区游离、吻合，利用腹壁 Trocar 的小切口取出标本，既保证肿瘤安全

彻底切除，同时避免腹壁辅助切口带来的困扰，美观、术后恢复可能更快，因而深受结直肠微创外科医师的推崇。尽管全腹腔镜结肠癌根治术在右半结肠癌中得到较多验证，但由于左半结肠癌发病率较低，远端横结肠、脾区、降结肠癌仅占大肠癌发病率的 2-5%，为众多腹腔镜结肠癌手术研究所排除，因此全腹腔镜左半结肠癌根治术仍缺乏高质量的临床研究。

因此我中心推动该研究来论证相关手术方式，验证相应手术方式的安全性、有效性，为后续临床实践提供更好的指导，最终使更广大结直肠癌患者受益。

二、研究过程

如果您决定参加这项临床研究。在进入研究前及研究过程中，医生将会询问您的病史及目前的状况，为您进行血液、心电图、CT、肠镜等检查。

本临床试验项目共包括全腹腔镜及腹腔镜辅助结肠癌根治术两种手术方案，如您同意参与试验，您将会通过随机接受其中的一种方案进行治疗。

（1）全腹腔镜左半结肠癌根治术：采用全腹腔镜技术，镜下完成系膜修剪，切除标本，并于腹腔镜下完成吻合，标本经脐部 Trocar 或右下腹 Trocar 小切口取出。吻合完成后均于镜下冲洗术区，留置引流管。

（2）腹腔镜辅助左半结肠癌根治术：采用传统腹腔镜辅助技术，于腹部正中或左腹直肌外缘采取小切口取出游离肠管，修剪系膜，切除标本，并完成吻合操作，将吻合后肠管送还腹腔，留置引流管。

参与本研究后，您在术后 30 天需按要求返院行 CT 检查及相关项目的复查。在手术结束后的约 5 年内，医生会通过查阅您的医疗记录或通过电话或其他方式，约 3-6 个月一次询问您或相关人员关于您的病情情况（包括治疗情况、预后情况等），收集您的临床病例资料。

注：

1） 医师将根据研究方案要求进行筛选，您可能因为某些病史及化验指标异常不能参加研究；

2） 您需要在治疗期间住院进行观察，由医师对您的治疗效果及安全性做出评价。

三、风险与不适

该研究是针对于我院拟行腹腔镜左半结肠癌根治术的前瞻性、随机对照、多中心研究，对于您来说，与我们进行沟通、交谈可能会有些心理不适。但该研究采用的手术方式均为腹腔镜结肠癌根治术，即微创手术方式，且均为临床常规手术，不会增加研究相关并发症。您在研究期间需要住院观察及采集相应数据，这些都可能因为占用您的时间而跟您工作或学习带来不便。您的研究医生会保证您不会受到不公正的待遇。

四、替代方案

参加本研究可能改善或不改善您的健康状况，您可以选择：

- 不参加本研究。
- 参加别的研究。
- 不接受任何治疗。

请与您的医生协商您的决定。

五、费用和收益

参与该研究，您不需要支付额外的费用。您也不会由于参与本研究而得到任何报酬。如果您同意参加本研究，您将有可能获得直接的医疗受益。对于结肠癌患者，全腹腔镜体内吻合可能降低切口感染率，减少术后并发症，从而降低围手术期死亡风险，改善患者短期预后。您为医学事业做出的贡献是非常有意义的。

疾病相关手术治疗以及相应的血液指标、病理指标检测等将由您承担。参加本项目不收取任何正常医疗以外的其他费用，不会给您增加额外的经济负担。

如果经正规评定出现了本研究造成您的人身损害的情况，课题组将支付您相应的医疗费用和根据法律法规进行补偿。

六、权益与隐私

通过对您的信息资料进行研究，将为您的治疗提供必要的建议，或为疾病的研究提供有益的信息。您参加本研究是自愿的，在研究过程中可随时与您的医生

交流。我们希望您能坚持完成本研究，但您仍有权可以选择不参加本研究或任何时候退出本研究，并不会因您的退出受到不公正的待遇以及影响您的正常治疗，研究医生会对您进行其他正常合理的治疗。

本研究中，如您发生严重不良事件，您将会得到及时积极的治疗。

如果您决定参加本项研究，您参加实验及在试验中的个人资料均属保密。负责研究医生及其他研究人员将使用您的医疗信息进行研究。这些信息可能包括您的姓名、年龄、地址、电话号码、病史，在不影响、损害您的正常诊疗，也不会危害您的健康前提下对的辅助检查结果进行采集及医学研究。这些研究结果发表时，将不会披露您个人的任何资料。

如果您需要其他治疗，或者您没有遵守研究计划，或者有任何其他原因，研究医生可以终止您继续参与本项研究。

您可随时了解与本研究有关的信息资料和研究进展，如果您有与本研究有关的问题，或您在研究过程中发生了任何不适与损伤，或有关于本项研究参加者权益方面的问题您可以与研究医师联系。

知情同意签署页

我已经阅读了本知情同意书。

我有机会提问而且所有问题均已得到解答。

我理解参加本项研究是自愿的。

我可以选择不参加本项研究，或者在任何时候通知研究者后退出而不会遭到歧视或报复，我的任何医疗待遇与权益不会因此而受到影响。

如果我需要其它治疗，或者我没有遵守研究计划，或者发生了与研究相关的损伤或者有任何其他原因，研究医生可以终止我继续参与本研究。

我将收到一份签过字的“知情同意书”副本。

受试者签名：______________________　　　　日期：____________

（注：如果受试者无行为能力/限制行为能力时，则需法定代理人签名和签署日期）

法定代理人签字：_________________　　　　日期：____________

（注：如果受试者不能阅读该知情同意书时，则需一名独立见证人证明研究者已将知情同意书的所有内容告知了受试者，独立见证人需签名和签署日期）

研究者声明：我确认已向您解释了本研究的详细情况，包括其权利以及可能的受益和风险，并给其一份签署过的知情同意书副本。

研究者签名：______________________　　　　日期：____________
