# Supplementary material for: Surgical site infection after intracorporeal anastomosis for left-sided colon cancer: study protocol for a non-inferiority multicenter randomized controlled trial (STARS)
Source: Trials. 2022 Nov 22;23:954. doi: 10.1186/s13063-022-06914-5 (PMC9682838; doi:10.1186/s13063-022-06914-5)
Supplement: Supplementary file 4 — Additional file 4. Informed consent in English. [file 13063_2022_6914_MOESM4_ESM.pdf]

Informed Consent

Trial：Surgical Site Infection after intracoporeal anastomosis for Left-sided Colon Cancer: study protocol for a non-inferiority multicenter Randomized Controlled Trial（STARS）

Protocol version: 1.0, 2019/12/20

Informed consent version:1.0, 2019/12/20

Research institution: The First Hospital of Jilin University

Principal investigator: Professor Wang Quan

Patient initials: ___________

You will be invited to participate in a clinical study, and this informed consent form provides you with some information to help you decide whether to participate in this clinical study. Please read it carefully, and if you have any questions, please ask the relevant physician in charge of the study.

Your participation in this study is voluntary. This study has been reviewed by the Ethics Committee of the First Hospital of Jilin University.

1. Research Background

Colorectal cancer is one of the most common malignant tumors in China. According to the latest cancer statistics, in 2015, the estimated new cases and deaths of colorectal cancer in our country were 376,000 and 191,000, respectively, ranking fifth in both incidence and mortality. With the advancement of medical science and technology, the treatment of colorectal cancer has become relatively mature, and a comprehensive treatment and individualized treatment mode based on surgery has been formed. The promotion of TME and CME surgical principles has greatly standardized colorectal surgery. The phase III COLOR II, COREAN, and CLASICC studies all showed no difference in local recurrence, disease-free survival, and overall survival between the two groups compared with open surgery. Laparoscopic colorectal cancer surgery has demonstrated safety and efficacy. While ensuring the curative effect of tumors, it has always been the goal of colorectal surgeons to strive to reduce the trauma of patients. In traditional laparoscopic-assisted surgery, a small incision in the abdominal wall is needed to trim the mesentery after dissection and dissociation in the cavity, and then the specimen is taken out and anastomosis is completed outside the abdominal wall.This can still cause some trauma to the patient, including postoperative pain and complications such as wound infection and incisional hernia.However, in some obese patients, the mesentery is thick and short, and the above operations are more difficult and even lead to the risk of mesentery tearing and bleeding, thus weakening the advantages of

laparoscopic surgery.Total laparoscopic left colon cancer radical resection is to complete the free and anastomosis of the operation area under the laparoscope, and use the small incision of Trocar in the abdominal wall to take out the specimen. This not only ensures complete tumor resection, but also avoids the trouble caused by auxiliary abdominal incision, so it is highly praised by colorectal minimally invasive surgeons. Total laparoscopic radical colonectomy has been validated in right colon cancer. However, due to the low incidence of left-sided colon cancer, the distal transverse colon, spleen region, and descending colon cancer only account for 2-5% of the incidence of colorectal cancer, which is excluded by many laparoscopic colon cancer surgery studies. There is still a lack of high-quality clinical research on colon cancer radical surgery. Therefore, our center promotes this study to verify the safety and efficacy of total laparoscopic surgery for left-sided colon cancer, and to provide better guidance for subsequent clinical practice, and ultimately benefit more colorectal cancer patients.

2. Research process

If you decide to participate in this clinical study, before entering the study and during the study, the doctor will ask about your medical history and current condition, and perform blood, electrocardiogram, CT, colonoscopy and other tests for you.

This clinical trial project includes two surgical options: total laparoscopy and laparoscopic-assisted radical colon resection. If you agree to participate in the trial, you will receive one of these options randomly for treatment.

(1) Total laparoscopic radical resection of left colon cancer: The mesentery was trimmed and excised under the microscope using the total laparoscopic technique, and the anastomosis was completed under the laparoscope. Specimens were taken out through the umbilical Trocar or the right lower quadrant trocar small incision. After the anastomosis was completed, the operation area was irrigated under the microscope, and the drainage tube was indwelled.

(2) Laparoscopic-assisted radical resection of left colon cancer: Using traditional laparoscopic-assisted techniques, a small incision was made in the middle of the abdomen or the outer edge of the left rectus abdominis to take out the free bowel and trim the mesentery, and the specimens were excised to complete the anastomosis. The anastomotic bowel was then returned to the abdominal cavity, and a drainage tube was placed in place.

After participating in this study, you will be required to return to the hospital for CT examination and re-examination of related items 30 days after the operation. Within about 5 years after the operation, the doctor will ask you or related personnel about your condition (including treatment, prognosis, etc.) by consulting your medical records or by telephone or other means about once every 3-6 months, and collect your clinical case data.

Note:

1) Physicians will screen according to the requirements of the research protocol, you may not be able to participate in the research due to some abnormal medical history and test indicators;

2) You need to be hospitalized for observation during the treatment period, and the doctor will evaluate your treatment effect and safety.

3. Risk and discomfort

This study is a prospective, randomized, controlled, multicenter study of laparoscopic left hemicolectomy in our hospital. It may be psychologically uncomfortable for you to communicate with us. However, the surgical methods used in this study were all laparoscopic minimally invasive surgery, and they were all routine clinical operations, which would not increase the complications related to the study. During the research period, you need to be hospitalized for observation and collect corresponding data, which may cause inconvenience to your work or study because of taking up your time. Your study doctor will ensure that you will not be unfairly treated.

4. Alternatives

Participating in this study may or may not improve your health. You may choose:

- Not participating in this study.
- Participate in other studies.
- Not receiving any treatment.

Please consult your decision with your doctor.

5. Costs and benefits

There is no additional fee for you to participate in this study, and you will not receive any payment for participating in this study. If you agree to participate in this study, you will likely receive direct medical benefits. For colon cancer patients, total laparoscopic surgery may reduce incision infection rates and postoperative complications, thereby reducing the risk of perioperative mortality and improving short-term patient outcomes. Your contribution to the medical career is very meaningful. Disease-related surgical treatment and the corresponding blood index, pathological index detection, etc. will be borne by you. Participating in this project does not charge any other fees other than normal medical treatment, and will not add additional financial burden to you. If the research has caused your personal injury through formal assessment, the research team will pay your corresponding medical expenses and make compensation according to laws and regulations.

6. Rights and Privacy

By researching your information, it will provide necessary advice for your treatment, or provide useful information for disease research. Your participation in this study is voluntary and you may communicate with your doctor at any time during the study.

We hope that you will continue to complete this study, but you still have the right to choose not to participate in this study or withdraw from this study at any time, and your withdrawal will not be unfairly treated and your normal treatment will not be affected.

In this study, if you have serious adverse events, you will receive timely and aggressive treatment.

If you decide to participate in this research, your participation in the experiment and your personal data in the trial will be kept confidential. The research doctor and other researchers will use your medical information to conduct research. This information may include your age, medical history, collection of auxiliary examination results and medical research under the premise that it will not affect your normal diagnosis and treatment and will not endanger your health. When these research results are published, no personal information about you will be disclosed.

The study doctor may terminate your continued participation in this study if you require additional treatment, or if you are not following the study plan, or for any other reason.

You can keep abreast of information and research progress related to this research. If you have questions about this study, or if you experience any discomfort or injury during the study, or have questions about the rights of participants in this study, you can contact the study physician.

Informed Consent Signing Page

I have read this informed consent form.

I had the opportunity to ask questions and all questions were answered.

I understand that participation in this study is voluntary.

I may choose not to participate in this study, or withdraw at any time by notifying the investigator without discrimination or retaliation, and my medical treatment and rights will not be affected.

The study doctor may terminate my continued participation in the study if I need additional treatment, or if I do not follow the study plan, or if a study-related injury occurs or for any other reason.

I will receive a signed copy of the Informed Consent Form.

Signature of Subject: _______________________ Date:_________________

(Note: If the subject is incapacitated/restricted, the signature and date of the legal representative are required)

Signature of Legal Representative: _____________ Date: ________________

(Note: If the subject cannot read the informed consent form, an independent witness is required to prove that the researcher has informed the subject of all the contents of the informed consent form, and the independent witness needs to sign and date)

Investigator Statement: I confirm that I have explained to you the details of this study, including its rights and possible benefits and risks, and have given him a copy of the signed informed consent.

Investigator Signature: _____________ Date: ________________
